# Thoracic injuries related to mechanical and manual cardiopulmonary resuscitation in the emergency department: a retrospective cohort study

**DOI:** 10.7717/peerj.20955

**Published:** 2026-03-24

**Authors:** Ömer Turan, Mehmet Ozgur Erdogan

**Affiliations:** 1 Forensic Sciences, Medeniyet University Hospital, Istanbul, İstanbul, Turkey; 2 Emergency Medicine, Bakirkoy Dr. Sadi Konuk Training and Research Hospital, Istanbul, Turkey

**Keywords:** Cardiopulmonary resuscitation, Device, Injury, Lung contusion

## Abstract

**Background:**

The aim of this study was to compare thoracic injuries associated with mechanical and manual cardiopulmonary resuscitation (CPR) methods.

**Methods:**

This single-center, retrospective observational cohort study included adult patients who underwent CPR and had post-resuscitation thoracic imaging and/or autopsy findings available. Demographic data (age, gender), type of CPR (mechanical or manual), duration of resuscitation, and thoracic injury patterns were extracted from hospital records. The recorded injuries included rib fractures, sternum fractures, clavicle fractures, scapula fractures, pulmonary contusions, pneumothorax, hemothorax, cardiac tamponade, myocardial hematoma or rupture, vertebral fractures, and mortality.

**Results:**

A total of 50 patients underwent mechanical CPR and 66 patients underwent manual CPR. CPR duration was similar between the mechanical and manual CPR groups (40.18 ± 4.83 *vs.* 41.10 ± 5.22 min; *p* = 0.330). Lung contusion was observed in 28% of the mechanical CPR group and in 9.1% of the manual CPR group, showing a statistically significant difference (*p* = 0.008). Rib fractures were more frequent with manual CPR (4.85 ± 1.64) compared with mechanical CPR (3.96 ± 2.29), with a statistically significant difference (*p* = 0.023). No statistically significant differences were found between the groups regarding sternum fractures, pneumothorax, hemothorax, pericardial effusion, or thoracic aortic hematoma.

**Conclusion:**

While rib fractures were more frequently seen in manual CPR, lung contusions were more commonly associated with mechanical CPR. These differences in injury patterns may have implications in determining the type of CPR administered during post-mortem examinations.

## Introduction

High-quality cardiopulmonary resuscitation (CPR) remains the strongest determinant of survival with favorable neurological outcomes following cardiac arrest (Liu et al., 2019). Mechanical chest compression devices have become increasingly utilized in both emergency department and prehospital settings (Sheraton et al., 2021; Senthilnathan et al., 2022). However, current resuscitation guidelines do not recommend their routine use (Soar et al., 2025; Berg et al., 2025). Instead, their application is reserved for situations in which high-quality manual compressions cannot be maintained, including patient transport, prolonged resuscitation efforts, cardiac catheterization procedures, and circumstances in which rescuer safety may be compromised. In such settings, mechanical devices are capable of delivering uninterrupted, guideline-compliant compressions that may improve hemodynamic stability (El-Menyar et al., 2024; Liu et al., 2019).

Both manual and mechanical CPR can result in thoracic trauma, including rib and sternal fractures, soft-tissue contusions, hemorrhage, and injuries to intrathoracic organs (Gao et al., 2021; Karasek et al., 2021). Emerging evidence suggests that mechanical compression systems may be associated with a higher incidence of lung contusions and parenchymal injury due to their uniform compression force (Kim et al., 2022; Karasek et al., 2022). These distinct trauma profiles hold important implications for forensic evaluation, as device-related injuries may be misinterpreted as unrelated traumatic or pathological findings if mechanical CPR use is not clearly documented.

Manual CPR remains subject to variability related to rescuer fatigue and technique differences (Erdogan et al., 2021; Orso et al., 2021). Mechanical devices, such as the LUCAS 2 system, aim to overcome these limitations by delivering standardized compressions through a backplate and automated piston arm (Liu et al., 2019).

Given these clinical and forensic considerations, a systematic comparison of thoracic injuries resulting from mechanical *versus* manual CPR is essential. The primary aim of this study was to compare the thoracic injury profiles resulting from mechanical *versus* manual CPR. The secondary aim was to identify distinctive injury characteristics—such as patterned soft-tissue findings, fracture distribution, and intrathoracic organ involvement—that may assist forensic experts in distinguishing mechanical CPR–related trauma from other causes of injury during post-resuscitation or post-mortem evaluation.

## Materials & Methods

### Study design and setting

This single-center, retrospective observational cohort study was conducted at the Emergency Department of Bakirkoy Dr. Sadi Konuk Training and Research Hospital. The study was approved by the University of Health Sciences Ethics Committee (No: 2024/01-14) and was carried out in accordance with the Declaration of Helsinki. Written informed consent was obtained from the patient’s relatives for the study.

### Study population

We analyzed adult patients (≥18 years) admitted for non-traumatic cardiac arrest between January 1, 2023, and January 1, 2024. Patients were eligible if they achieved return of spontaneous circulation (ROSC) and underwent thoracic or upper abdominal computed tomography (CT) within 6 h after resuscitation. Exclusion criteria included traumatic cardiac arrest, missing clinical data, absence of post-ROSC CT imaging, nondiagnostic CT quality, or age <18 years. All patients were intubated, mechanically ventilated, and maintained in the supine position during transport and CT imaging. All cardiac arrest cases included in the study occurred within the hospital setting. Therefore, all events were witnessed by medical personnel, and cardiopulmonary resuscitation was initiated immediately by trained healthcare staff. As a result, there were no unwitnessed arrests or bystander-initiated CPR cases in this cohort.

### Resuscitation methodology

Patients were stratified into two groups according to the resuscitation method utilized:

1. Mechanical CPR, performed using the LUCAS 2™ chest compression system (Jolife AB/Stryker, Lund, Sweden).

2. Manual CPR, delivered by trained emergency personnel in accordance with the most recent American Heart Association (AHA) 2020 Guidelines and European Resuscitation Council (ERC) 2021 Guidelines.

Resuscitation duration, method, and device application details were obtained from standardized CPR documentation forms.

### CT imaging protocol and injury definition

All CT examinations were performed using a multi-detector CT scanner with standard thoracic trauma imaging parameters. Axial, coronal, and sagittal reconstructions were evaluated.

Thoracic and upper abdominal injuries were assessed using established radiologic trauma criteria. Lung contusion was defined as a non-segmental parenchymal opacity characterized by patchy or confluent ground-glass or consolidative densities without air bronchograms, appearing within hours after blunt chest compression, located in regions consistent with external mechanical force, and not attributable to cardiac dysfunction or fluid overload (Rodriguez et al., 2006; Huber-Wagner et al., 2009).

To differentiate lung contusion from pulmonary edema, radiologists relied on the absence of interlobular septal thickening, lack of smooth septal lines, absence of diffuse vascular congestion, and the presence of focal or asymmetric lesions aligned with compression zones.

The following injury categories were systematically evaluated: rib, sternal, clavicular, scapular, and vertebral fractures; pulmonary contusions or lacerations; pneumothorax or hemothorax; cardiac tamponade; myocardial hematoma or rupture; mediastinal emphysema or vascular injury; and upper abdominal organ injury when present.

Performing CT imaging within six hours after ROSC allows documentation of acute CPR-related injuries before the development of post-resuscitation or post-mortem artifacts such as hypostasis, evolving edema, or fluid overload. This early imaging serves as an objective baseline record that can later be correlated with autopsy findings. As such, the CT scans obtained in this time frame provide valuable information for medico-legal evaluation by helping forensic specialists distinguish mechanical CPR–related injuries from unrelated traumatic or pathological lesions.

### Data collection and outcome measures

Data collection encompassed demographic variables (age, sex), resuscitation parameters (CPR method, duration), and thoracoabdominal injuries including rib, sternum, clavicle, scapula, and vertebral fractures; pulmonary contusions; pneumothorax; hemothorax; cardiac tamponade; myocardial hematoma or rupture; and in-hospital mortality. CT images were independently reviewed by two blinded radiologists using standardized injury classification systems. The primary outcome was the comparison of thoracic injury profiles between mechanical and manual CPR. The secondary outcome was the identification of distinctive injury characteristics that could help forensic specialists differentiate mechanical CPR-related trauma from injuries unrelated to resuscitation.

### Power analysis

A *post-hoc* power analysis was conducted using G*Power software (version 3.1.9.2). For a one-tailed, independent samples *t*-test with group sizes of 50 and 66, an alpha error probability of 0.05, and a medium effect size (*d* = 0.5), the study demonstrated high statistical power (0.84, or 84%) to detect a significant difference between groups.

### Statistical analysis

Statistical analysis was performed using SPSS v25.0 (IBM Corp., Armonk, NY, USA). Descriptive statistics presented continuous variables as mean ± standard deviation or median with interquartile range (IQR), while categorical variables were expressed as frequencies (%). Normality of distribution was assessed using both the Kolmogorov–Smirnov and Shapiro–Wilk tests. Group comparisons employed independent *t*-tests for normally distributed continuous variables, Mann–Whitney *U* tests for non-parametric data, and Pearson’s *χ*^2^ or Fisher’s exact tests for categorical variables. A two-tailed *p*-value <0.05 was considered statistically significant.

## Results

This study included a total of 116 patients. Diabetes mellitus was present in 37 patients (31.9%), hypertension in 67 (57.8%), coronary artery disease in 40 (34.5%), cerebrovascular disease in 20 (17.2%), chronic kidney disease in 18 (15.5%), malignancy in 14 (12.1%), and chronic obstructive pulmonary disease (COPD) in 26 patients (22.4%).

Among the 116 patients included in the analysis, heart failure–associated arrest represented the leading presumed etiology (*n* = 33, 28.4%), followed by respiratory failure (*n* = 30, 25.8%) and intracranial events (*n* = 20, 17.2%). Ischemia-related causes accounted for 13 cases (11.2%), while arrhythmia-related mechanisms were identified in 11 patients (9.5%). Regarding pre-arrest cardiac rhythm, sinus tachycardia was the most common finding (*n* = 35, 30.2%), followed by atrial fibrillation with rapid ventricular response (*n* = 26, 22.4%) and sinus bradycardia (*n* = 18, 15.5%) (Table 1).

**Table 1:** Presumed cause of cardiac arrest and initial cardiac rhythm distribution.

**Presumed cause of cardiac arrest**	**n**	**%**
Heart failure–associated cardiac arrest	33	28.4
Intracranial event–related cardiac arrest	20	17.2
Respiratory failure	30	25.8
Arrhythmia-related cardiac arrest	11	9.5
Ischemia-related cardiac arrest	13	11.2
Sepsis	14	12.1
Pulmonary embolism	3	2.6
Unknown medical cause	6	5.2
**Pre-arrest cardiac rhythm**		
Sinus Tachycardia	35	30.2
Sinus Bradycardia	18	15.5
AF-CVR	5	4.3
AF-RVR	26	22.4
AF -SVR	12	10.4
Nodal (junctional) rhythm	7	6.0
Second- or third-degree AV block	5	4.3
SVT	4	3.4
VT	4	3.4

**Notes.**

AF-CVR Atrial Fibrillation (AF) with controlled ventricular response AF-RVR AF with rapid ventricular response AF-SVR AF with slow ventricular response SVT Supraventricular Tachycardia VT Ventricular Tachycardia AV Atrioventricular

There was no statistically significant difference in mean age between the manual CPR group (72.95 ± 5.01 years; *n* = 66) and the mechanical CPR group (73.20 ± 6.15 years; *n* = 50) (*p* = 0.819). There was no statistically significant difference in CPR duration between the mechanical CPR group (40.18 ±  4.83 min) and the manual CPR group (41.10 ± 5.22 min; *p* = 0.330); Table 2. There was no statistically significant difference in gender distribution between the manual and mechanical CPR groups, with females comprising 27 (40.9%) patients in the manual CPR group and 22 (44.0%) patients in the mechanical CPR group (*p* = 0.739).

**Table 2:** Comparison of continuous variables between mechanical and manual CPR groups.

**Variable**	**Mechanical CPR (Mean ± SD)**	**Manual CPR (Mean ± SD)**	*P*-value
Age (years)	72.95 ± 5.01	73.20 ± 6.15	0.819
CPR duration (min)	40.18 ± 4.83	41.10 ± 5.22	0.330
Number of rib fractures	4.85 ± 1.64	3.96 ± 2.29	0.023

**Notes.**

Values are presented as mean ± standard deviation (SD).

Student’s *t*-test was used for comparisons of continuous variables.

Fourteen (28%) patients had lung contusion in mechanical CPR group and 6 (9.09%) patients had lung contusion in manual CPR group. Lung contusion was statistically significantly higher (*p* = 0.008) in mechanical CPR group. Sternum fracture, pneumothorax, hamemothorax, paericardial effusion, thoracic aortic haematoma had no statistical difference between mechanical CPR and manual CPR groups. Aortic rupture, liver injury and gastrointestinal system (GIS) perforation was not diagnosed in study groups. Corresponding data are presented in Table 3. Rib fractures occurred more frequently in the manual CPR group (4.85 ± 1.64) than in the mechanical CPR group (3.96 ±2.29), and this difference reached statistical significance (*p* = 0.023) (Table 2).

**Table 3:** Comparison of injury types between mechanical and manual CPR groups.

Injury type	**Mechanical CPR (*n* = 50)**	**Manual CPR (*n* = 66)**	*P*-value
Lung contusion	14 (28.0%)	6 (9.1%)	0.008^*^
Unilateral	2 (4.0%)	1 (1.5%)	
Bilateral	12 (24.0%)	5 (7.6%)	
Sternum fracture	12 (24.0%)	27 (40.9%)	0.056^*^
Pneumothorax	7 (14.0%)	12 (18.2%)	0.547^*^
Hemothorax	9 (18.0%)	11 (16.7%)	0.851^*^
Pericardial effusion	3 (6.0%)	3 (4.5%)	0.726^#^
Aortic rupture	0	0	^–^
Thoracic aortic hematoma	1 (2.0%)	0	0.431^#^
Liver injury	0	0	^–^
Gastrointestinal perforation	0	0	^–^

**Notes.**

Values are presented as number of cases (percentage).

* Chi-square test was used.

# Fisher’s exact test was applied when expected cell counts were <5.

– indicates that statistical comparison was not applicable due to zero frequency in both groups.

## Discussion

Resuscitation research has advanced more slowly compared with many other medical disciplines, because ethical and logistical constraints hinder randomized intervention trials in cardiac arrest (Mentzelopoulos et al., 2018). Mechanical CPR devices represent one of the most significant recent advances in resuscitation practice, as they standardize chest compression quality and have seen rapidly expanding adoption in both hospital and prehospital settings (Soar et al., 2025; Berg et al., 2025). Their equivalence to manual CPR in survival outcomes supports this widespread implementation (Kim et al., 2022; Azeli et al., 2022; Chun et al., 2023). However, the capacity of mechanical devices to deliver sustained, effective compressions may increase CPR-related thoracic injuries (Saleem et al., 2022).

Moreover, mechanical and manual CPR techniques may produce distinct thoracic injury patterns, and recent literature increasingly supports a more differentiated understanding of these effects (Petrovich et al., 2023). Prior studies have documented higher rates of pulmonary contusion with mechanical devices, attributed to their constant depth and force of compression (Karasek et al., 2022; Kim et al., 2022). Experimental and imaging-based studies have shown that mechanical chest compressions may promote greater alveolar-capillary stress, regional overdistension, and edema formation compared with manual CPR (Magliocca et al., 2021; Magliocca et al., 2024; Rezoagli et al., 2022). The present study aligns with these findings, demonstrating a significantly greater incidence of lung contusion in patients who underwent mechanical CPR. This association may reflect the inability of device-driven compressions to adjust to individual variations in chest wall compliance, underlying parenchymal vulnerability, or anatomic positioning—factors that trained providers may modulate during manual CPR. Importantly, CPR duration did not differ significantly between the mechanical and manual CPR groups in our cohort, suggesting that the observed difference in lung contusion rates is unlikely to be driven by resuscitation time alone. This is clinically relevant, as prior translational and imaging-based studies have demonstrated that prolonged CPR duration is independently associated with greater lung injury severity, including CPR-associated lung edema, underscoring the need to account for CPR duration when interpreting pulmonary injury patterns (Magliocca et al., 2021). Conversely, manual CPR in our cohort produced a higher number of rib fractures compared with mechanical CPR, consistent with reports that manual compressions generate more variable and sometimes excessive focal pressure due to fatigue, technique differences, or inconsistent hand positioning (Berkowitz, Pleimann & Rosenfeld, 2018; El-Menyar et al., 2024). Similar observations in large registries have noted that while mechanical compression systems standardize force delivery, manual CPR frequently results in higher fracture burden, supporting the pattern seen in our results.

No significant differences were observed in sternal fractures, pneumothorax, hemothorax, or major vascular injury, paralleling prior investigations suggesting that these outcomes are less influenced by compression modality and more by arrest characteristics or baseline frailty (Chun et al., 2023; Kim et al., 2022). Importantly, neither group demonstrated catastrophic complications such as aortic rupture or solid-organ laceration, supporting the notion that these events remain rare after CPR regardless of technique.

The forensic implications of these findings warrant emphasis. As described in recent medico-legal literature, recognizing patterned CPR-related trauma is critical for differentiating iatrogenic injury from true traumatic or pathological lesions (Karasek et al., 2022). Our observation that mechanical CPR predominantly yields pulmonary contusion, whereas manual CPR more commonly causes rib fractures, provides imaging markers that may support both post-resuscitation evaluation and post-mortem interpretation—particularly when device use is undocumented.

This study has several limitations. Its single-center, retrospective design and relatively small sample size (*n* = 116) may introduce selection bias and limit generalizability. Importantly, the analysis was restricted to CT findings in patients who achieved ROSC; therefore, we were unable to evaluate fatal cases that typically undergo autopsy. This absence of post-mortem correlation is a major limitation, as deaths may involve more extensive or qualitatively different CPR-related injuries that are not captured by imaging alone. Additionally, only the LUCAS 2 mechanical CPR device was examined, limiting applicability to other systems. Variability in rescuer technique during manual CPR could not be fully controlled. Finally, the lack of detailed pre-arrest pulmonary comorbidity data limits contextual interpretation of lung contusion susceptibility. Future multi-center studies integrating autopsy data and standardized CPR provider training would yield a more comprehensive understanding of injury mechanisms.

## Conclusions

In this cohort, mechanical and manual CPR were associated with different thoracic injury patterns, with mechanical CPR more frequently demonstrating pulmonary contusions and manual CPR showing a higher prevalence of rib fractures. These observations may assist clinicians and forensic specialists in better interpreting post-resuscitation imaging findings. Nevertheless, the results should be interpreted cautiously given the study’s retrospective design and the absence of autopsy data, and further research is needed to validate these patterns in broader patient populations.

## Supplemental Information

10.7717/peerj.20955/supp-1 Supplemental Information 1 CONSORT checklist

10.7717/peerj.20955/supp-2 Supplemental Information 2 Raw data

## Additional Information and Declarations

### Data Availability

The following information was supplied regarding data availability: The raw data is available in the Supplementary File.
